# Chronic rhinosinusitis due to cyano-acrylic glue after endoscopic transsphenoidal pituitary surgery

**DOI:** 10.1186/s12893-020-00866-w

**Published:** 2020-09-16

**Authors:** Majid Bani-Ata, Firas Alzoubi, Bashar Abuzayed, Ala”a A. Alhowary, Abdelwahab J. Aleshawi

**Affiliations:** 1 Department of Special Surgery, Division of Otolaryngology, Faculty of Medicine, Jordan University of Science and Technology, P.O.Box: 3030, Irbid, 22110 Jordan; 2 Department of otolaryngology, Faculty of Medicine, Jordan University of Science and Technology, Irbid, 22110 Jordan; 3 Gardens Hospital, Al Sab Bin Jathamah St, P.O.Box: 4144, Amman, Jordan; 4 Department of Anesthesia, Faculty of Medicine, Jordan University of Science and Technology, Irbid, 22110 Jordan; 5 Department of Special Surgery, Division of Ophthalmology, Faculty of Medicine, Jordan University of Science and Technology, Irbid, 22110 Jordan

**Keywords:** Acrylic glue, Transsphenoidal approach, Chronic rhinosinusitis

## Abstract

**Background:**

To reduce the risk of cerebrospinal fluid leak, clinicians utilize a filling material placed in the sella followed by floor reconstruction with various materials, including glue sealing. Cyano-acrylic glue Glubran®2 glue is commercially available and is generally used as embolizing agent and for the prevention of cerebrospinal fluid leakage.

**Case Description:**

A 25-year-old woman underwent endoscopic endonasal transsphenoidal surgery for pituitary adenoma. After tumor resection, sellar floor reconstruction was performed by mucosal graft and Glubran®2 glue. The early post-operative period was uneventful. However, 2 months after surgery, the patient complained of headache, facial pain and greenish foul-smelling nasal discharge with solid particles dripping from the nose. Medical treatment was unsuccessful. Brain MRI showed inflammation and thickening of the sphenoidal and para-sphenoidal mucosa. The patient underwent endoscopic endonasal surgery and a solid glass-like mass surrounded by inflamed infected mucosa was seen in the inferior and lateral aspects of the sphenoid sinus. Efforts were made to erupt and de-crust the solid mass until total resection was achieved. Early post-operative period was uneventful, and a course of antibiotics was continued until total disappearance of the discharge.

**Conclusion:**

To the best of our knowledge, this is the first case reporting of acrylic glue (Glubran®2)-related sinusitis. Surgeon should be aware about similar side effects for the glue material that would complicate the surgery.

## Background

Cerebrospinal fluid (CSF) leaks are considered serious complications in transsphenoidal pituitary surgery and efficient tissue sealing is an important preventive measure. For reducing the risk of CSF leaks, clinicians have attempted various methods of sellar reconstruction. These typically utilize a filling material (i.e., fat, collagen sponge) placed in the sella followed by floor reconstruction with various materials, including glue sealing [1]. Cyano-acrylic glue Glubran®2 glue (GEM Srl, Viareggio, Italy) is commercially available and is generally used as a fixative for injured tissue, for hemostasis, as embolizing agent and for the prevention of CSF leakage [2–4]. We have been using this agent routinely in duroplasty after transsphenoidal pituitary surgery for the past 5 years, without any recorded glue-related complications. In this report, we present an unusual case of cyano-acrylic glue- induced chronic rhinosinusitis after endoscopic endonasal pituitary surgery. To the authors’ best knowledge, this is the first report of cyano-acrylic glue (Glubran®2)- related chronic rhinosinusitis.

## Case Presentation

A 25-year-old woman was referred to our outpatient clinic with complaints of chronic headache and irregular menses. Hormone profile showed only moderate increase in Prolactin (96 ng/ml). Sellar magnetic resonance imaging (MRI) showed a pituitary mass. The patient had total pituitary adenectomy using an endoscopic endonasal transsphenoidal approach. After tumor resection, diaphragmatic opening was seen with intra-operative evidence of CSF leak. Sellar floor reconstruction was performed by mucosal graft and Glubran®2 glue filling the surgical cavity. Early post-operative period was uneventful and clinical and histopathologic finding were consistent with a non-functional pituitary adenoma.

**Fig. 1:**
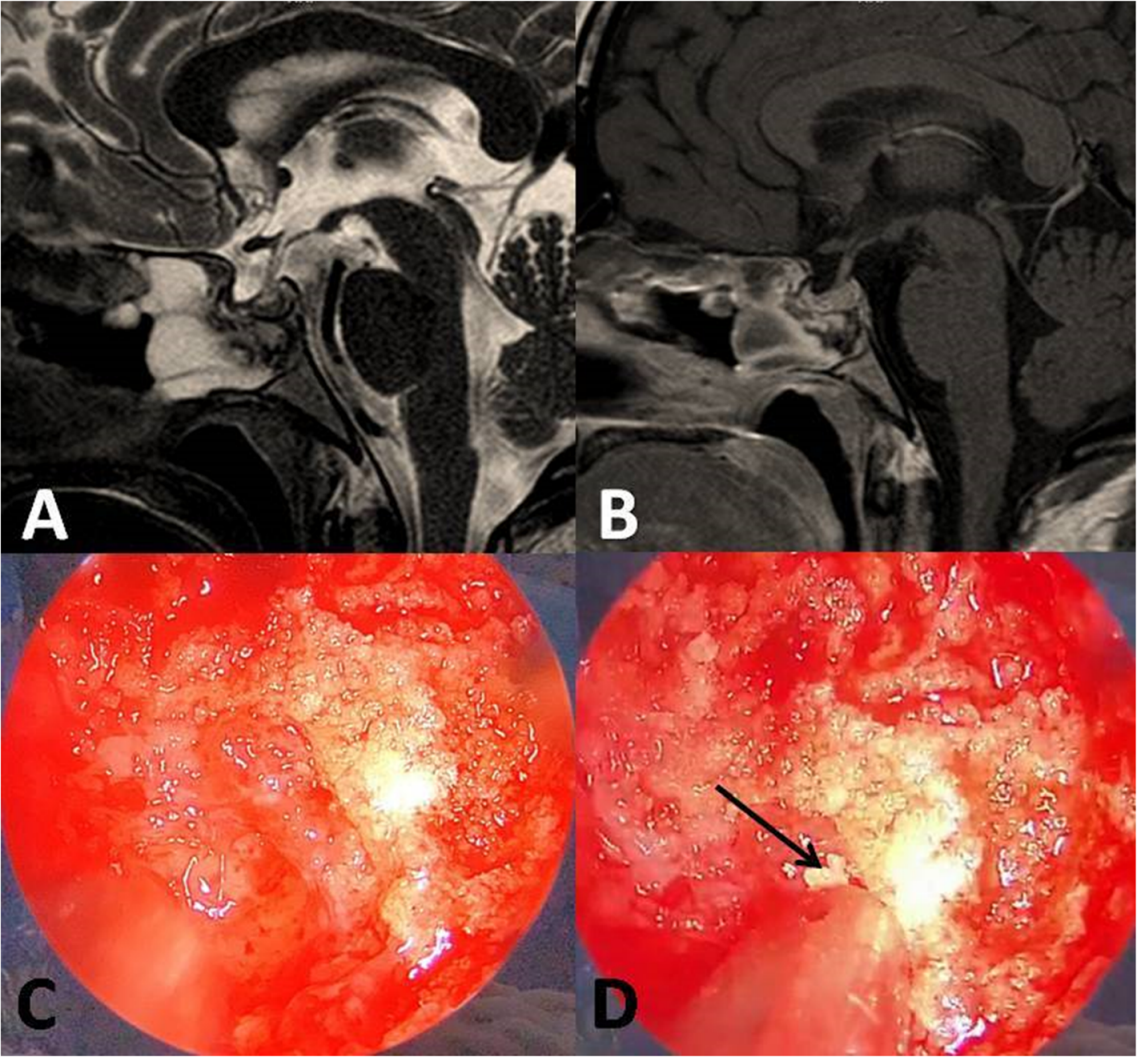
**a**) T2-weigthed and **b**) post-contrast sagittal brain MRI showing inflammation and thickening of the spheniodal and para-sphenoidal mucosa. **c** and **d**) Intraoperative endoscopic view showing the solid glass-like mass (acrylic glue) was seen in the inferior and lateral areas to the sphenoid sinus surrounded with inflamed infected mucosa. De-crustation of the mass shows fragmentation of the mass with glass-like particles *(arrow)*

After 2 months of surgery, the patient complained of headache, facial pain and greenish foul-smelling nasal discharge with solid particles. Patient was diagnosed with rhinosinusitis and treated with multiple courses of nasal decongestants and antibiotics for 4 months but without improvement. Brain MRI showed inflammation and thickening of the sphenoidal and para-sphenoidal mucosa (Fig. 1 a and b).

Surgery with endoscopic endonasal approach was decided. Intra-operatively, the sellar floor was seen intact with no CSF leak nor discharge. A solid glass-like patches (acrylic glue) was seen in the inferior and lateral areas of the sphenoid sinus and was surrounded with inflamed infected mucosa and abundant pussy discharge (Fig. 1 c and d). Efforts were made to erupt and de-crust the solid mass until total resection was achieved. Early post-operative period was uneventful, and a course of antibiotics was continued until total regression of the discharge. Endoscopic follow-up was performed in the 1^st^, 2^nd^ and 3^rd^ post-operative months, and showed no signs of rhinosinusitis with well-healed nasal mucosa.

## Discussion and conclusion

Cyano-acrylates are synthetic glues utilized as adhesive materials in the operations because of their efficient bond power and rapid-onset action. The general chemical structure of the cyanoacrylate molecule is CH2 = C (CN)CO2R, with R representing an organic molecular group in the variable alkyl group [5]. Cyano-acrylates are working by forming long chains of liquid monomers called cyanoacrylic esters that; in the existence of anionic molecules; polymerase with each other. These chains gain the adhesive ability and constitute a solid film over an surgical application surface [5].

In turn, Glubran 2® is a type of cyanoacylate glue but it differs from other cyanoacylate glues currently available (i.e. Histoacryl) as it is composed of the mixture of two monomers rather one simple monomer [3]. This make the Glubran 2® a comonomer which is formed by N butyl 2 cyanoacrylate (NBCA) and MS monomer. NBCA is a cyanoacylate monomer commonly found in many other glues [3].

The mixing of MS to the basic monomer achieves a reduction in the toxicity of the basic monomer by two factors: polymerization of the NBCA at 45° C in an exothermic reaction with a slightly higher polymerization time than. In fact, the longer the chain of the cyanoacrylate is, the less cytotoxic adhesion [3]. The use of Glubran®2 has been expanded as a duroplasty glue [2], Omentopexy and abdominal hernia fixation [6], bronchopleural fistula sealing [7], for decreasing alveolar air leak in thoracic surgery [8] and brain tumors embolization. [4]. In our center, we routinely use Glubran®2 for sealing the reconstruction of sellar floor after transsphenoidal pituitary surgery.

The use of commonly available cyano-acrylates adhesives may be associated with multiple complications, one of which is toxicity. Initially, tissue toxicity was attributed to the release of heat from the exothermic reaction of polymerization. However, subsequently, the toxicity was attributed to the degradation of their alkyl chains into cyanoacetate and formaldehyde, which results in slower metabolism and elimination with subsequent tissue accumulation and inflammation [5]. Increasing number of carbon molecules in the alkyl chains would slow the degradation and therefore reduce the accumulation of toxic compounds, as in the case of Glubran®2 [5]. Although intra-operative findings in our surgery showed well-healed sellar floor without CSF leak or local reaction to the glue, it would appear that leakage of the glue into the nasal mucosa has induced the inflammation and caused clinical rhinosinusitis observed in our patient. As the patient’s dura was relatively unaffected compared to the healthy nasal mucosa, it is likely that different tissues may demonstrate different reactions to the glue. Why it is not fully evacuated or aspirated prior the completion of the surgery, this is due to the formation of firm ceramic-based plug after glue application which makes it very difficult to aspirate from the sphenoid sinus. Thus, careful local application is very important in order to avoid wide spreading of the glue over the mucosa. Our recommendations to decrease the risk of this complication is to limit the application of the glue only on the sellar floor and to apply maneuvers to prevent leaking of the glue into the nasal cavity, such as pin point single drops application to the duroplasty site with lowering the head position to prevent gravity related glue leaking into the nasal cavity.

Comparing to the cyanoacylate glue, fibrin sealants; including TISSEEL (Baxter, Deerfield, IL, USA); have been commercially available since the 1990s. They consist of human-derived fibrinogen and thrombin and are classified as thrombogenic hemostats designed to treat low level (not severe) brisk arterial or venous bleeding [9]. The disadvantage of fibrin sealants includes time-consuming preparation. Furthermore, there have been reports that fibrin sealants can precipitate acute immune responses; chronically, they are associated with adhesion formation and infection [9]. In addition, the fibrin sealants can be used as an adhesive material for another type of operations [10]. The polyethylene glycol-based hydrogel sealant; as DuraSeal (Covidien); which is biocompatible, self-polymerizing, nontoxic, water-soluble, absorbable, flexible, and strongly adherent to tissue has been recently used to avoid CSF leak complications; and there has been no reported sealant-related adverse events in the literature [11]. It also showed lower postoperative CSF leak incidence than fibrin glue [12]. BioGlue surgical adhesive sealant, is a mixture of purified bovine serum albumin (45 %) and glutaraldehyde (10 %) [13]. Due to the confirmed advantages of DuraSeal over Glubran®2, we recommend the using DuraSeal when available. Glubran®2 can be reserved in cases which other preferred glue options are not available with careful local application as described previously. The bovine serum comes from North American cattle herds that are free from transmissible bovine spongiform encephalopathy. It is purified by heat precipitation, chromatography, and then gamma-irradiation [13]. The advantage of BioGlue is that the bifunctional glutaraldehyde molecule covalently bonds the bovine serum albumin molecules to each other, and to lysine in proteins on the cell surface and in proteins in the extracellular matrix [13]. However, many various complications after the use of BioGlue have been described in the literature, including failure of sealing, infection and mass effect formation [13].

In conclusion, Cyano-acrylic glue Glubran®2 glue is widely used fixative agent for injured tissue, for hemostasis, as embolizing agent and as a sealant for prevention of CSF leakage. In this report, we present an unusual case of cyano-acrylic glue- induced chronic rhinosinusitis after endoscopic endonasal pituitary surgery. To the authors’ best knowledge, this is the first report of cyano-acrylic glue (Glubran®2)- related chronic rhinosinusitis.

## Data Availability

Not applicable.

## Abbreviations

CSF: Cerebrospinal fluid
NBCA: N butyl 2 cyanoacrylate
